# Flavonoids in Kidney Health and Disease

**DOI:** 10.3389/fphys.2018.00394

**Published:** 2018-04-24

**Authors:** Félix Vargas, Paola Romecín, Ana I. García-Guillén, Rosemary Wangesteen, Pablo Vargas-Tendero, M. Dolores Paredes, Noemí M. Atucha, Joaquín García-Estañ

**Affiliations:** ^1^Departamento de Fisiología, Facultad de Medicina, Instituto de Investigación Biosanitaria GRANADA, Hospitales Universitarios de Granada, Universidad de Granada, Granada, Spain; ^2^Departamento de Fisiología, Facultad de Medicina, Instituto Murciano de Investigación Biomédica, Universidad de Murcia, Murcia, Spain; ^3^Departamento de Ciencias de la Salud, Area de Fisiología, Universidad de Jaén, Jaén, Spain

**Keywords:** kidney function, acute kidney injury, chronic kidney disease, flavonoids, nephroprotection, diabetes mellitus, arterial hypertension

## Abstract

This review summarizes the latest advances in knowledge on the effects of flavonoids on renal function in health and disease. Flavonoids have antihypertensive, antidiabetic, and antiinflammatory effects, among other therapeutic activities. Many of them also exert renoprotective actions that may be of interest in diseases such as glomerulonephritis, diabetic nephropathy, and chemically-induced kidney insufficiency. They affect several renal factors that promote diuresis and natriuresis, which may contribute to their well-known antihypertensive effect. Flavonoids prevent or attenuate the renal injury associated with arterial hypertension, both by decreasing blood pressure and by acting directly on the renal parenchyma. These outcomes derive from their interference with multiple signaling pathways known to produce renal injury and are independent of their blood pressure-lowering effects. Oral administration of flavonoids prevents or ameliorates adverse effects on the kidney of elevated fructose consumption, high fat diet, and types I and 2 diabetes. These compounds attenuate the hyperglycemia-disrupted renal endothelial barrier function, urinary microalbumin excretion, and glomerular hyperfiltration that results from a reduction of podocyte injury, a determinant factor for albuminuria in diabetic nephropathy. Several flavonoids have shown renal protective effects against many nephrotoxic agents that frequently cause acute kidney injury (AKI) or chronic kidney disease (CKD), such as LPS, gentamycin, alcohol, nicotine, lead or cadmium. Flavonoids also improve cisplatin- or methotrexate-induced renal damage, demonstrating important actions in chemotherapy, anticancer and renoprotective effects. A beneficial prophylactic effect of flavonoids has been also observed against AKI induced by surgical procedures such as ischemia/reperfusion (I/R) or cardiopulmonary bypass. In several murine models of CKD, impaired kidney function was significantly improved by the administration of flavonoids from different sources, alone or in combination with stem cells. In humans, cocoa flavanols were found to have vasculoprotective effects in patients on hemodialysis. Moreover, flavonoids develop antitumor activity against renal carcinoma cells with no toxic effects on normal cells, suggesting a potential therapeutic role in patients with renal carcinoma.

## Introduction

Renal disorders are among the most common diseases. Acute kidney injury (AKI) is associated with a greater long-term risk of cardiovascular disease (CVD) and chronic kidney disease (CKD) (Van Berendoncks et al., 2010). There is a lack of effective treatment against CKD, which is increasingly prevalent worldwide and related to a higher likelihood of CVD and death (Coresh et al., 2007). There is therefore major clinical and social interest in the development of therapies to treat renal disorders. The present review addresses the effects of flavonoids on renal physiology and their renoprotective effects in nephropaties of different origin.

**Figure 1:**
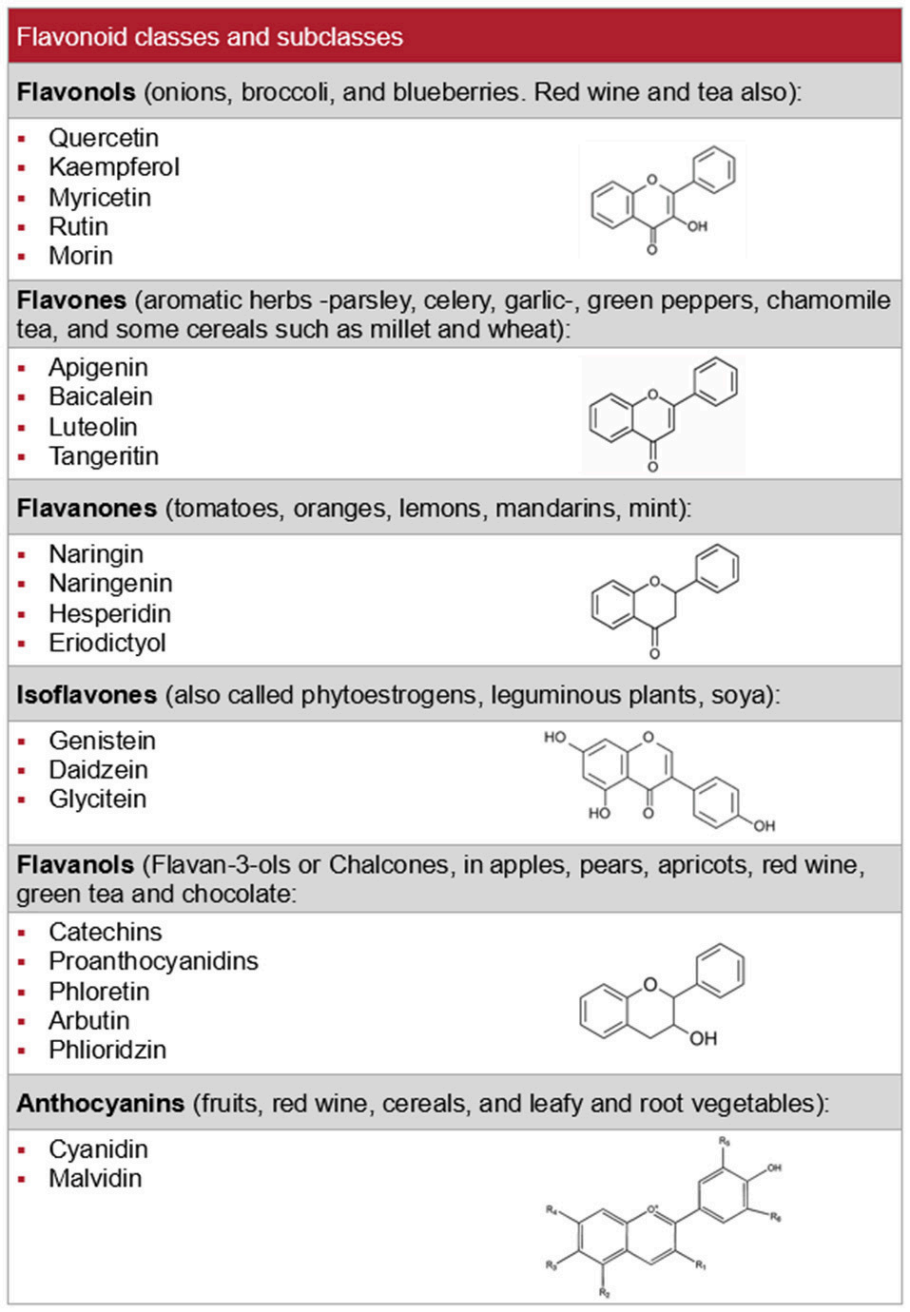
Flavonoid classes, their main subclasses and typical sources.

The term flavonoids refers to thousands of plant compounds with a common basic structure, phenylchromane, which allows the generation of multiple flavonoid subclasses (Figure 1) including flavonols, flavones, catechins, anthocyanidins, isoflavones, dihydroflavonols, and chalcones (Middleton et al., 2000). Variable amounts of these compounds are found in vegetables, fruits, nuts, spices, herbs, red wine and tea, among others (Williams et al., 2004). Flavonoids are one of the main classes of polyphenols, which have numerous pharmacological activities, exert antioxidant effects (Middleton et al., 2000; Manach et al., 2004), and are known to promote cardiovascular health, but less is known about their role in renal function and disease. We provide an update on knowledge of the effects of flavonoids on renal function and the action mechanisms involved, especially in relation to the most frequent acute and chronic nephropathies encountered in clinical practice. We also review preclinical data on the protective effect of flavonoids against renal fibrosis and on their anti-tumor activity.

## Renal physiology

**Figure 2:**
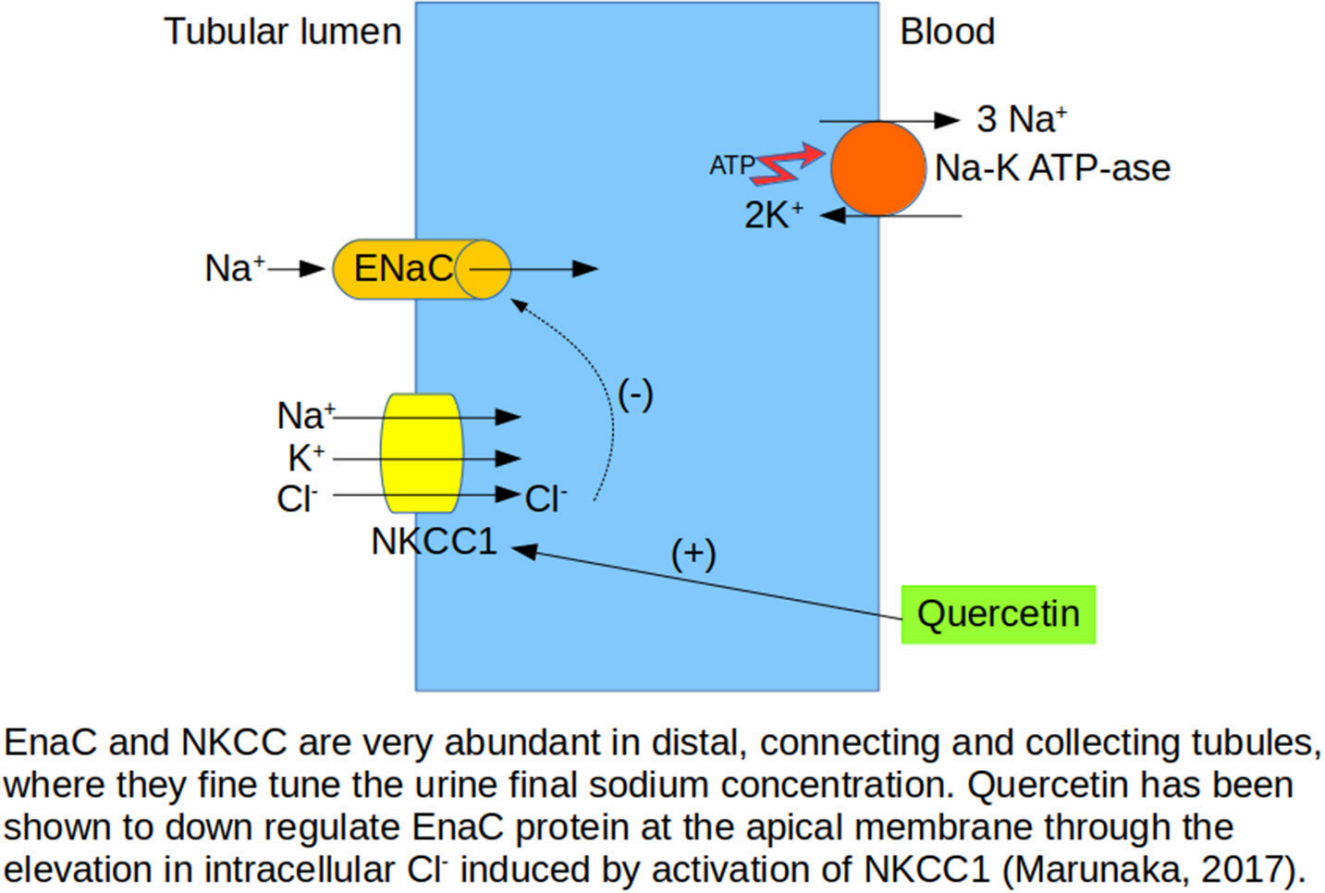
Effects of quercetin on sodium transporters in kidney tubules affect sodium balance.

Quercetin downregulates the renal expression of epithelial Na^+^ channel (ENaC) in hypertensive Dahl salt-sensitive rats, and this effect is associated with a reduction in systolic blood pressure (Aoi et al., 2004). The ENaC plays a key role in the kidney, regulating Na^+^ reabsorption in renal tubules, and quercetin also stimulates the Na^+^-K^+^-2Cl^−^ cotransporter 1 (NKCC1), a key ion transporter regulating cytosolic Cl^−^ concentration (Marunaka, 2017). NKCC1 activation affects several body and cellular functions, such as renal Na^+^ reabsorption, thereby regulating the concentration and volume of extracellular fluid (Figure 2). Renal Na,K-ATPase is an important regulator of sodium homeostasis in the organism, and its activity is enhanced in spontaneously hypertensive rats (SHRs). Mezesova et al. (2010) reported that quercetin reduced Na,K-ATPase activity and affinity to the sodium binding site in both normotensive and hypertensive rats. de Almeida et al. (2017) observed that nothofagin, a dihydrochalcone isolated from Leandra dasytricha leaves, had diuretic, natriuretic and potassium-sparing effects in both normotensive and hypertensive rats, finding these effects to be associated with increased prostanoid generation, muscarinic receptor activation, and antioxidant properties. Polymethoxylated flavones extracted from Rubus rosaefolius Sm. (Rosaceae) and orally administered to male Wistar rats also demonstrated diuretic and natriuretic activity (de Souza et al., 2017) related to prostaglandin production. In summary, quercetin and other flavonoids appear to modify several factors that increase water and sodium excretion, decreasing blood volume, and these changes likely contribute to their antihypertensive actions, besides their well-known cardiovascular effects.

## Hypertensive nephropathy

Arterial hypertension is associated with renal dysfunction and is one of the main etiologies of renal disease in the developed world. A key objective of the treatment of arterial hypertension, besides the reduction in blood pressure, is renal protection. The antihypertensive effects of the flavonoid quercetin are well documented in various animal models of hypertension by Duarte and colleagues. In 2001, it was reported to reduce the elevated blood pressure and renal hypertrophy of SHRs in parallel with a reduction in their systolic blood pressure, with no changes in WKY controls. These effects were attributed to the antioxidant properties of the drug (Duarte et al., 2001), and the results were later confirmed in other laboratories (Romero et al., 2010). In the DOCA-salt rat model, dietary quercetin was found to reduce systolic blood pressure and renal hypertrophy and improve kidne**y** glutathione transferase activity (Galisteo et al., 2004a), potassium depletion, and oxidative stress (Galisteo et al., 2004b). Hence, quercetin demonstrates not only antihypertensive but also renal antioxidant properties in this model. Epicatechin treatment for 5 weeks also prevented the increased systolic blood pressure and the proteinuria of DOCA-salt rats and produced a reduction in systemic oxidative stress and endothelin-1 markers (Gómez-Guzmán et al., 2012). Similar effects on these variables were observed after treatment with red wine polyphenols in the same model of hypertension (Jiménez et al., 2007). Chronic treatment with quercetin also reduced systolic blood pressure and proteinuria in the two-kidney one-clip hypertension Goldblatt model, while having no effect on control rats (García-Saura et al., 2005). In the same model, Kaur and Muthuraman (2016) found that rutin, a flavonoid glucoside, reduced blood pressure, renal hypertrophy, plasma renin content, and tissue TBARS and increased GSH levels.

The antihypertensive and renal protective effects of quercetin have also been evaluated in the L-NAME model, with an exhaustive analysis of the histological changes (Duarte et al., 2002). Chronic NO synthesis inhibition induces not only high blood pressure but also renal hypertrophy and proteinuria and intense histological lesions of the kidney; these include hyaline arteriopathy and vascular wall thickening, with a moderately reduced lumen, and an increase in oxidative stress. Quercetin strongly inhibited the hypertension induced by L-NAME and prevented the renal hypertrophy, vascular lesions, and proteinuria, suggesting that this flavonoid also has a protective effect against renal injury caused by the chronic inhibition of NO synthesis.

SHRs treated with a grape seed proanthocyanidin extract for 22 weeks exhibited a dose-dependent reduction in inflammatory infiltration at the renal interstitium and a decrease in albuminuria but failed to attenuate the increase in blood pressure (Wang et al., 2015). However, Lan et al. (2015) reported that grape seed proanthocyanidins had an anti-hypertensive effect in DOCA-salt hypertensive rats, preventing renal injury as well as improving proteinuria, renal hypertrophy, and renal fibrosis and oxidative stress markers *via* attenuation of renal JNK and p38 kinase activities. Another flavonoid, morin, was found to bring hypertension and renal function markers/histopathology findings to near-normal levels in DOCA-salt hypertensive rats (Prahan et al., 2012).

In fructose-fed hypertensive rats, Palanisamy and Venkataraman (2013) observed that genistein produced beneficial effects by lowering blood pressure, preserving renal ultrastructural integrity and promoting eNOS activation and NO synthesis in renal cells.

Flavonoids have also been administered in combination with calcium antagonists and angiotensin-converting enzyme-inhibitors. Puerarin is an isoflavone found in a number of plants and herbs, such as the root of pueraria (radix puerariae). The combination of felodipine with puerarin improved blood pressure and heart rate more effectively and enhanced protective effects against renal interstitial fibrosis in renovascular hypertensive rats (Bai et al., 2013). In humans, the effects of pycnogenol in combination with the angiotensin-converting enzyme inhibitor ramipril have been evaluated in hypertensive patients with early signs of renal dysfunction (Cesarone et al., 2010). Their joint administration for 6 months lowered albuminuria, serum creatinine, and C-reactive protein and increased kidney cortical flow to a greater degree than ramipril alone. The results of these two studies indicate that the combination of flavonoids with classic antihypertensive drugs may be a useful therapeutic approach, especially in patients with uncontrolled hypertension.

This section has reported evidence that flavonoids prevent or ameliorate renal injury associated with arterial hypertension. The action mechanism may be secondary to the reduction of blood pressure and to the well-known protective effects of flavonoids in the vascular system. However, flavonoids may also act directly on the renal parenchyma and interfere with signaling pathways that can affect the development of renal injury (e.g., oxidative stress, inflammatory molecules, protein kinases, and matrix metalloproteinases), regardless of their effects on blood pressure.

## Obesity and diabetes

High fructose consumption has been associated with renal alterations that play a role in CKD development. Prince et al. (2016) reported that (-)-epicatechin given in the rat chow diet of male Sprague Dawley rats prevented or ameliorated the adverse effects of high fructose consumption (10% w/v fructose in drinking water) for 8 weeks. Fructose-fed rats showed proteinuria, decreases in nephrin, synaptopodin, and Wilms' tumor transcription factor (WT1) in the renal cortex, and all podocyte dysfunction indicators, associated with an increase in oxidative stress markers, changes in NO synthase (NOS) activity and expression, and an increase in pro-inflammatory factors. All of these renal abnormalities were ameliorated by dietary supplementation with (-)-epicatechin.

Immoderate fat accumulation causes both oxidative stress and inflammation, which can induce kidney damage in obese subjects. Eo et al. (2017) studied the renal protective effects on high-fat diet obese mice of different dosages of ecklonia cava polyphenol extract given by gavage for 12 weeks, finding that its administration lowered inflammation and oxidative stress. In addition, supplementation with this polyphenol extract significantly up-regulated renal SIRT1, PGC-1α, and AMPK, which are associated with renal energy metabolism, thereby improving the aberrant renal energy metabolism induced by a high-fat diet.

Diabetic nephropathy is a progressive kidney disease and is the main microvascular complication in diabetes. It has been reported that a central role in the progression of diabetic nephropathy is played by oxidative stress, inflammation, and fibrosis. Streptozotocin (STZ)-induced type 1 diabetes is the most frequently used murine model in studies of diabetic nephropathy, and its utilization has revealed several flavonoids that exert important renoprotective effects by various mechanisms. Thus, Tian et al. (2015) reported that diabetic nephropathy could be ameliorated in STZ-induced diabetic rats by icariside II treatment through an increase in endothelial cell contents and downregulation of the TGF-β/Smad/CTGF signaling pathway and oxidative stress levels. These beneficial effects appear to be mediated by its antioxidant capacity and the recruitment of endogenous EdU+ progenitor cells to the kidney tissue. Malik et al. (2017) found that apigenin treatment attenuated renal dysfunction, oxidative stress, and fibrosis in STZ-induced diabetic rats and prevented MAPK activation, inhibiting inflammation and apoptosis, with renal tissue histopathological findings of a reduction in inflammation, collagen deposition, and glomerulosclerosis.

The isoflavonoid puerarin, from the Chinese medicinal herb Radix puerariae, protected against diabetic nephropathy in STZ-induced diabetic mice (Xu et al., 2016), improving blood urea nitrogen/serum creatinine, fasting blood glucose, and 24 h urine protein levels as well as kidney tissue damage, with a reduction in mitochondrial damage. The authors (Xu et al., 2016) affirmed that puerarin exerts its renal protection by attenuating the SIRT1/FOXO1 pathway.

Podocyte injuries (e.g., slit diaphragm effacement) are associated with the presence of albuminuria in diabetic nephropathy. Using a STZ-induced diabetic nephropathy mouse model, Zhang et al. (2016) examined the effect of hyperoside, the 3-O-galactoside of quercetin, on proteinuria and renal damage at an early stage. They found that 30 mg/kg/day of oral hyperoside for 4 weeks decreased urinary excretion of microalbumin and glomerular hyperfiltration. Hyperoside also improved glomerular mesangial matrix expansion, podocyte process effacement, and slit diaphragm reduction, and it restored nephrin and podocin levels in diabetic nephropathy mice. All of these data indicate the beneficial effects of hyperoside against renal damage and podocyte injury.

Naringenin, a flavonone most frequently obtained from grapefruit, orange, or tomato peel/skin, ameliorated STZ-induced diabetic rat renal impairment by downregulating TGF-β1 and IL-1 through the modulation of oxidative stress (Roy et al., 2016). Moreover, naringenin-treated diabetic rats revealed an improved renal histology and a significant reduction in apoptotic activity.

Flavonoids have also shown renoprotective effects in type 2 diabetic mice (Db/db). Thus, Db/db mice orally treated with the leaf extract (150 mg/kg/day) of Moringa oleifera for 5 weeks reduced the histopathological damage and the expression in renal tissue of tumor necrosis factor-alpha (TNF-α), interleukin (IL)-1β, IL-6, cyclooxygenase-2, and inducible NOS (Tang et al., 2017). Epigallocatechin-3-gallate, extracted from green tea, also reduced renal pathology alterations and delayed diabetic kidney disease progression in diabetic db/db mice through the suppression of hyperglycemia-induced oxidative stress (Yang et al., 2016).

Damage to capillaries in the glomeruli is the first step in the development of diabetic nephropathy. Endothelial dysfunction is an early marker of diabetic CVD and may play a role in progressive diabetic nephropathy, while hyperglycemia-induced endothelial hyperpermeability is crucial for this disease. Wang X. et al. (2016) assessed the effects of rutin pretreatment in an *in vitro* model of hyperglycemia-induced barrier dysfunction using human renal glomerular endothelial cells. They reported the prevention of hyperglycemia-disrupted renal endothelial barrier function *via* the RhoA/ROCK signaling pathway through a reduction in reactive oxygen species mediated by nuclear factor E2-related factor-2 (Nrf2) activation (Wang X. et al., 2016).

In summary, the oral administration of flavonoids has been found to counter the adverse renal effects in mice or rats of high fructose consumption, high-fat diet, STZ-induced type1 diabetes, and type 2 diabetes (Db/db mice) and to prevent hyperglycemia-disrupted renal endothelial barrier function. These effects are associated with a reduction in oxidative stress and pro-inflammatory factors which attenuated urinary microalbumin excretion and glomerular hyperfiltration through the improvement of podocyte injury. The latter considered an important factor for the development of albuminuria in diabetic nephropathy.

## Nephrotoxic agents

Severe AKI can be produced by sepsis in bacterium-infected patients, especially in the intensive care setting, and various flavonoids have been tested against lipopolysaccharide (LPS)-induced renal inflammation. The water extract of Heterobathmia diffusa, a flavonoid-rich herb used in traditional Chinese medicine, has been shown to have a protective effect on this inflammation in mice, inhibiting production of TNF-α, IL-1β, IL-6, and monocyte chemoattractant protein (MCP)-1 and enhancing IL-10 production in serum and tissues (Ye et al., 2015). Male Sprague Dawley rats pretreated for 4 days with dietary (-)-epicatechin prevented the adverse effects of LPS challenge by inhibiting TLR4 upregulation and NO activation and the resulting downstream events, such as NF-kB activation (Prince et al., 2017). LPS-mediated nephrotoxicity was also reduced by pre-treatment with luteolin, a bioactive flavonoid, through an improvement in renal oxidant status and a reduction in NF-κB activation and inflammatory and apoptotic factors (Xin et al., 2016).

Nephrotoxicity is one of the main adverse effects of chemotherapy with cisplatin, widely used as a first-line drug against various solid cancers, involving the generation of reactive oxygen species, inflammation, apoptotic pathway stimulation and MAPK pathway activation. Histopathology study showed that cisplatin treatment was responsible for severe renal necrosis/degeneration, tubular hyaline casts, intertubular hemorrhage, glomerular congestion/swelling, and leukocyte infiltration of renal tissue, limiting its dosage and clinical usefulness (Ma et al., 2017). It is therefore vital to develop anti-cancer drugs that protect the kidney against toxicity. Several flavonoids attenuate cisplatin-induced renal injury in rats and mice, mitigating cisplatin-induced histopathologic alterations and reducing the increases in serum creatinine and blood urea nitrogen. Their mechanism of action involves several pathways of the inflammatory cascade and oxidative perturbations (Tanabe et al., 2012; Malik et al., 2015, 2016; Arab et al., 2016; Chao et al., 2016; He et al., 2016; Hassan et al., 2017; Huang D. et al., 2017; Huang Y. C. et al., 2017; Lee et al., 2017; Ma et al., 2017), including: the downregulation of activated NF-κB p65 protein expression and its downstream effectors (e.g., iNOS and TNF-α), with restoration of the anti-inflammatory IL-10; and reductions in phospho-NF-κB p65 and phospho-P38 MAPK activation and Nrf2 expression in cisplatin-induced renal injury. Flavonoids also downregulated the expression of the apoptotic marker caspase-3, inhibiting cisplatin-induced apoptosis and thereby favoring renal cell survival. Mouse studies have also revealed a reduction in mitochondrial mass, oxidative phosphorylation complexes, and superoxide dismutase levels in cisplatin nephropathy, which was prevented by the administration of flavanol (-)-epicatechin without modifying the anticancer effect of cisplatin in HeLa cells (Tanabe et al., 2012). Besides offering renal protection against cisplatin nephrotoxicity, some flavonoids have also demonstrated antitumor activity. Thus, the cytotoxic actions of cisplatin in Hep3B and HCT-116 human cancer cell lines were enhanced by tangeretin (Arab et al., 2016), while COLO205 and HeLa tumor cell growth was inhibited by puerarin, which dose-dependently promoted the antitumor activity of cisplatin in these tumor cells (Ma et al., 2017). Finally, a study in rats found that methotrexate-induced renal damage, apoptosis, and oxidative stress were ameliorated by quercetin (Erboga et al., 2015). It is interesting to note that flavonoids may exert both anticancer and renoprotective actions in chemotherapy.

Several other flavonoids have shown renal protective effects against numerous nephrotoxic agents that frequently cause acute or chronic kidney injury. The general and common mechanism of action of flavonoids is their attenuation of renal oxidative stress and inflammation. Flavonoids that have been reported to protect rat or mouse kidney against nephrotoxic agents include: flavocoxid (El-Kashef et al., 2015), pinocembrin (Promsan et al., 2016), and gossypin (Katary and Salahuddin, 2017), which attenuated gentamicin-induced nephrotoxicity in rats; trigonella foenum-graecum (TFG) seeds (Pribac et al., 2015), which prevented cell deterioration and improved renal morphology and function in the kidney of alcoholized rats; hydroxyflavone and 7-hydroxyflavone, which protected against nicotine-induced oxidative stress (Sengupta et al., 2017); baicalin, which protected against lead-induced renal oxidative damage in mice (Zhang et al., 2017); and epigallocatechin-3-gallate, which attenuated cadmium-induced chronic renal injury/fibrosis (Chen et al., 2016) and reduced contrast-induced renal injury alone (Gao et al., 2016) or with glycerol-induced renal damage (Palabiyik et al., 2017).

## Ischemia-reperfusion injury and other surgical procedures

A major cause of kidney failure is renal ischemia/reperfusion (I/R) injury, which can occur in kidney transplantation, partial nephrectomy, renal artery angioplasty, aortic aneurysm surgery, and elective urological surgery, among others. I/R injury triggers a cascade of events that produce injury to renal cells and their eventual death. Renal reperfusion injury is associated with increased mortality and morbidity due to AKI. In rat studies, renal I/R is usually associated with increased serum creatinine, urea and uric acid, elevated diuresis and natriuresis alongside impaired concentrating ability and reduced renal superoxide dismutase, catalase, and glutathione activities. The histopathological changes induced by I/R are characterized by glomerular injury and extensive tubular damage, with signs of tubular cell swelling, tubular dilatation, interstitial edema, and moderate-to-severe necrosis (Bhalodia et al., 2009; Lin et al., 2014; Zhao et al., 2016; Yang et al., 2017). Flavonoids and other phytochemical compounds have shown marked protective effects against I/R-induced AKI, generally mediated by their antioxidant and anti-inflammatory actions.

Diosmetin is a flavonoid glycoside that inhibits the renal inflammatory response and cellular apoptosis (Yang et al., 2017) and was found to protect against I/R-induced AKI in mice by suppressing nuclear factor-κB and mitochondrial apoptosis pathways and activating the nuclear factor erythroid 2-related factor 2/heme oxygenase-1 pathway. The traditional Chinese herb root baicalin has been reported to protect against renal I/R injury by inhibiting the production of proinflammatory cytokines, including TNF-α and IL-1β (Lin et al., 2014). Flavonoids from Rosa laevigata Michx fruit also exerted nephroprotective effects against I/R AKI by suppressing oxidative stress and inflammation, affecting the Sirt1/Nrf2/NF-κB signaling pathway (Zhao et al., 2016). Other antioxidants that have shown protective effects against I/R-induced renal damage in rats include the extract of Benincasa cerifera fruit, widely consumed in India and other tropical countries (Bhalodia et al., 2009), and aqueous garlic extract, whose administration 15 min before ischemia and immediately before reperfusion decreased I/R-induced injury in rat kidney (Kabasakal et al., 2005; Bagheri et al., 2011). AKI induced by cardiopulmonary bypass was found to be improved in diabetic rats by the preoperative oral administration of epigallocatechin-3-gallate, a major component of the polyphenolic fraction of green tea, thanks to its antioxidant properties (Funamoto et al., 2016). This treatment has been proposed for prophylactic renal protection against AKI after cardiopulmonary bypass in the clinical setting, especially in high-risk diabetic patients.

## Chronic kidney disease

CKD is a major and increasing health problem worldwide, and there is no effective treatment. Flavonoids from different sources, alone or in combination with stem cells, have significantly improved kidney function in several models of chronic renal failure.

**Figure 3:**
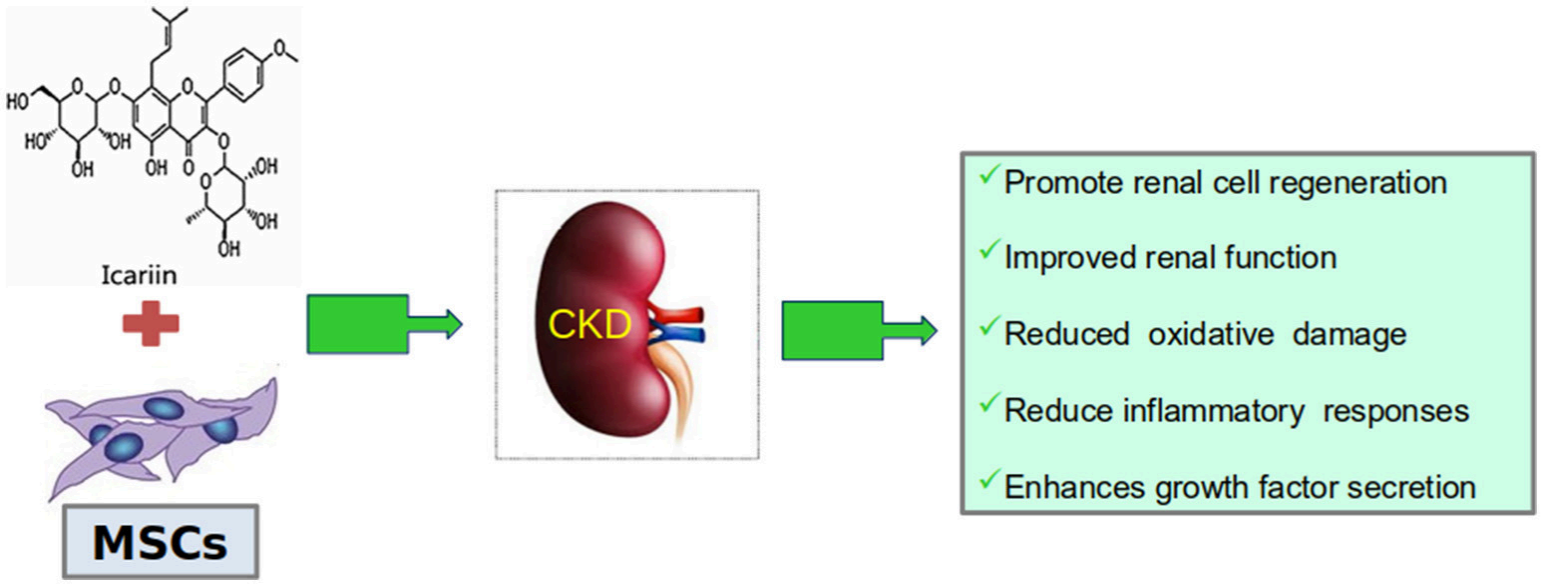
Icariin together with mesenchymal stem cells (MSCs) improve a rat model of CKD.

Daily doses for 8 weeks of icariin, a kidney-tonifying active flavonoid component of the Epimedium plant species, widely used in Chinese medicine, protected against renal function impairment and histological abnormalities secondary to chronic renal failure produced by 5/6 nephrectomy (Huang et al., 2015). Icariin also reduced TGF-β1 expression, increased CD24, CD133, Osr1, and Nanog expressions, and augmented the number of CD133(+)/CD24(+) renal stem/progenitor cells, considered responsible for these protective effects. In a later study (Figure 3), administration of icariin combined with human umbilical cord mesenchymal stem cells to rats with chronic kidney failure improved the kidney function, reduced the oxidative damage, inflammatory responses and fibrosis levels, and enhanced the expression of growth factors (Li et al., 2017).

The multi-organ dysfunctions produced by systemic lupus erythematosus generate a severe immune regulation disorder, with autoantibody overproduction, lupus nephritis, aberrant CD4+ T cell activation, and immune complex-mediated inflammation. Quercitrin, a glycoside formed from the flavonoid quercetin, diminished serum antibodies, reduced CD4+ T cell activation, and ameliorated lupus nephritis symptoms in a systemic lupus erythematosus mouse model (Li et al., 2016). Liao et al. (2016) also reported that icaritin, an active extract of the genus Epidemium used in traditional Chinese medicine, inhibited the over-activation of CD4+ T cells from systemic lupus erythematosus and improved renal damage in mice.

In a rat study Ali et al. (2016), the flavonoid chrysin, which exerts strong antioxidant and anti-inflammatory activities, was tested on adenine-induced CKD, administering doses of 10, 50, or 250 mg/kg 10 consecutive days after the consumption of adenine (0.25%, w/w) for 35 days. Chrysin, especially at 250 mg/kg, mitigated all manifestations of adenine-induced renal dysfunction, improved creatinine clearance, and reduced concentrations of urea, creatinine, plasma neutrophil gelatinase-associated lipocalin, urinary N-Acetyl-beta-D-glucosaminidase, uremic toxin indoxyl sulfate, and some inflammatory cytokines. Histopathological markers of renal inflammation and fibrosis and renal oxidative stress were also improved.

Naringenin, a natural flavonoid with various biological and pharmacological properties attenuated cardiac remodeling and cardiac dysfunction in a rat model of cardiorenal syndrome (Liu et al., 2016), improving renal function and reducing serum creatinine, blood urea nitrogen, type-B natriuretic peptide, aldosterone, angiotensin II, C-reactive protein, and urine protein concentrations as well as decreasing ROS levels through an increase in nuclear factor erythroid 2-related factor 2 expression.

Finally, a double-blind, randomized, placebo-controlled trial in humans reported that dietary cocoa flavanols had a vascular protective effect in patients on hemodialysis (Rassaf et al., 2016).

## Renal apoptosis and fibrosis

Apoptosis is an active response of cells to altered microenvironments and is characterized by cell shrinkage, chromatin condensation, and DNA fragmentation. In kidney cells, apoptosis has been observed in renal epithelial cells, endothelial cells, mesangial cells, and podocytes, among other cell types. Hyperglycemia is among microenvironmental factors that may facilitate apoptosis, which is a key trigger of diabetic nephropathy. Mohan et al. (2017) explored the capacity of epigallocatechin-3-gallate to prevent apoptosis in rats on a high-fat diet and with STZ-induced diabetic nephropathy, demonstrating an improvement in renal function by the downregulation of TGF-β, ameliorating their diabetic nephropathy. Baicalin, a flavonoid widely used to treat infectious and inflammatory diseases, also inhibited renal cell apoptosis in a mouse model of sepsis produced by cecal ligation and puncture (Zhu et al., 2016) through inhibition of the pro-apototic regulator BAX. Rats with gentamicin-induced kidney injury were also protected from oxidative stress-induced apoptosis by aqueous extracts of Rhizoma smilacis glabrae through inhibition of proapoptotic caspase-3 activation (Liu et al., 2017).

Renal fibrosis plays an important role in the pathogenesis of renal failure and in the progression of CKD. Epithelial to mesenchymal transition (EMT) contributes to the renal accumulation of matrix proteins and therefore to progressive renal fibrosis. Flavonoids have demonstrated activity against unilateral ureteral obstruction, 5/6 nephrectomy, hypertension, and diabetes, among other nephropathies. Renal injury induced by unilateral ureteral obstruction is frequently used in a rat model of renal fibrosis, and Wang B. et al. (2016) found that daily oral gavage (100 mg/kg) for 2 weeks of rutin, a polyphenolic flavonoid, ameliorated their kidney interstitial fibrosis, reducing renal interstitial injury and suppressing interstitial collagen deposits. Rutin decreased macrophage infiltration, proinflammatory cytokine expression, and phosphorylation of nuclear factor-κB p65 and inhibited extracellular matrix accumulation by reducing type I/III collagen and fibronectin expressions. Rutin also decreased α-smooth muscle actin expression and maintained E-cadherin expression, preventing EMT in renal tubular cell. The authors attributed these changes to anti-inflammatory action and TGF-β1/Smad3 signaling inhibition. Rutin also improved the proteinuria and renal oxidative stress in 5/6-nephrectomized rats, reducing glomerulosclerosis and tubulointerstitial injuries (Han et al., 2015). The treatment of mice with unilateral ureteral obstruction using the isoflavone puerarin, found in a number of plants and herbs such as the root of Pueraria (Radix puerariae), reduced renal fibrosis through the inhibition of oxidative stress induced-epithelial cell apoptosis *via* MAPK signaling (Zhou et al., 2017). In this same model, intraperitoneal administration of baicalin every 2 days for 10 days ameliorated renal fibrosis and suppressed EMT *via* inhibition of TFG β1 production and Smad2/3 phosphorylation signaling (Zheng et al., 2017).

Hypertension-induced renal fibrosis is an important factor in the progression of hypertensive nephropathy. A 4-week course of dietary apigenin, an anti-hypertensive flavone abundant in celery, reduced DOCA-salt-induced structural and functional kidney damage and fibrosis, with a reduction in the expressions of TFG β1, the Smad2/3 signaling pathway, and extracellular matrix proteins (Wei et al., 2017).

Kang et al. (2015) investigated the effects of chrysin (5,7-dihydroxyflavone), found in herbs and bee propolis, on EMT and tubulointerstitial fibrosis due to chronic hyperglycemia in human renal proximal tubular epithelial cells. These were incubated in media with high levels of glucose in the absence and presence of 1–20 μM chrysin for 72 h. Chrysin inhibited the glucose-induced renal EMT, tubular cell production of collagen and collagen fiber deposition. Taken together, the data reported in this section indicate that several flavonoids possess antifibrotic properties and that their main action mechanisms are EMT inhibition and interference with TGF-β1/Smad signaling.

## Renal carcinoma

Flavonoids have demonstrated action against several types of cancer cells. Thus, apigenin exerts antitumor activity in various cancers. An evaluation by Meng et al. (2017) of its effects and action mechanisms in renal carcinoma cells found that it induced DNA damage, G2/M phase cell cycle arrest, p53 accumulation, and apoptosis, which together inhibited ACHN RCC cell proliferation *in vitro* and *in vivo*. Apigenin is a highly promising agent against renal carcinoma due to these antitumor effects and low *in vivo* toxicity. Woo and Kwon (2016) reported that jaceosidin, a flavonoid isolated from artemisia vestita, exerted anti-tumor and anti-proliferative activities in many cancer cells and produced apoptosis in different human renal carcinoma cells (Caki, ACHN, A498, and 786-O), with the activation of caspase-3 and cleavage of poly (ADP-ribose) polymerase. This treatment also reduced mitochondrial membrane potential and Bax activation, releasing cytochrome C into the cytosol. Interestingly, apoptosis was not induced by Jaceosidin in normal human umbilical vein cells.

*In vitro* and *in vivo* studies have demonstrated the anticancer capabilities in renal cell carcinoma cells of silibinin, an antioxidant flavonoid extracted from milk thistle, which inhibited renal cell carcinoma growth through caspase-dependent apoptosis (Ma et al., 2015). In an *in vivo* mouse xenograft model, silibinin also reduced tumor growth, specifically targeting the mTOR-GLI1-BCL2 signaling pathway.

Galangin, a flavonoid extracted from the root of Alpinia officinarum was reported to possess antiproliferative action and to inhibit renal cell carcinoma (786-0 and Caki-1) invasion by suppressing EMT, inducing apoptosis and producing ROS in renal carcinoma cells (Cao et al., 2016). Han et al. (2016) observed that the combination of galangin and TRAIL significantly induced apoptosis in renal carcinoma (Caki, ACHN, and A498) but not in normal mouse kidney cells or human normal mesangial cells, suggesting the potential utilization of galangin as a sensitizer of TRAIL-resistant cancer cell therapy. Meng et al. (2015) combined quercetin with anti-sense oligo gene therapy (Snail gene inhibition) and found that each suppressed the proliferation and migration of Caki-2 cells, inducing cell cycle arrest and apoptosis, with a strong suppression of renal carcinoma cells being achieved by their combination. Their findings supported this combination of natural product and gene therapy as a novel treatment for renal cancer.

**Figure 4:**
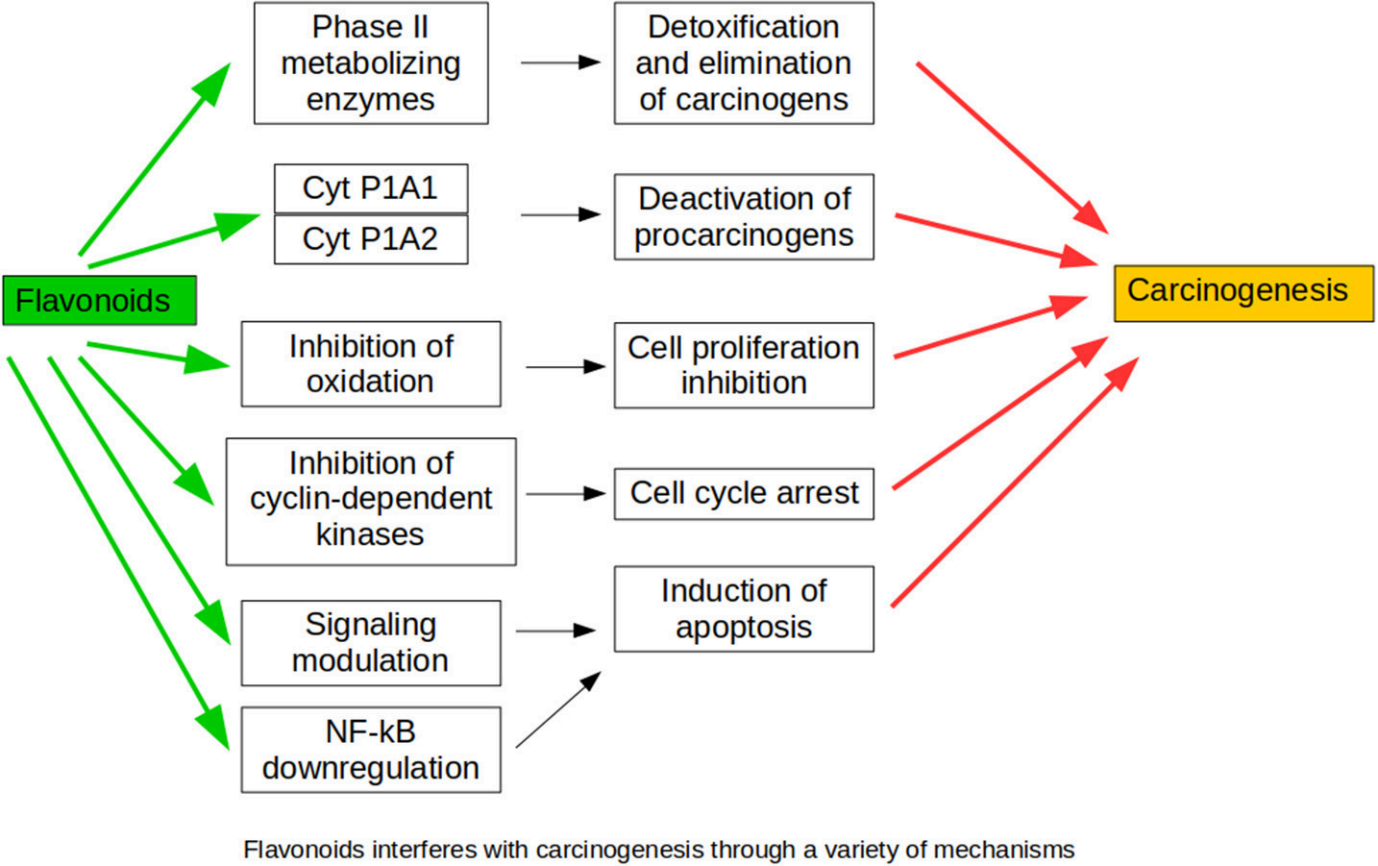
Mechanisms through which flavonoids interfere with carcinogenesis mechanisms.

The above findings demonstrate that flavonoids develop antitumor activity against renal carcinoma cells without toxic effects on normal cells, suggesting their potential value to treat renal carcinoma alone or in combination (Figure 4).

## Undesirable effects of flavonoids

Green tea is a popular beverage with protective effects against several renal diseases. Thus, polyphenols from this product demonstrated protective activity against renal injury in lead-exposed rats through ROS scavenging activity (Wang H. et al., 2016) and against renal ischemia-reperfusion injury in rats (Lv et al., 2015). They also protected diabetic rats against AKI after cardiopulmonary bypass (Funamoto et al., 2016) and attenuated high-fat diet-induced renal oxidative stress (Yang et al., 2015), as well as showing hypouricemic effects (Chen et al., 2015). However, despite its positive effects on these types of kidney disease, green tea may also have undesirable effects in chronic renal failure. Green tea is rich in catechins, which are putative substrates of anion transporters such as OAT1 and OAT3. Chronic renal failure is associated with high levels of indoxyl sulfate and *p*-cresyl sulfate, nephro-cardiovascular toxins whose renal excretion is mediated by organic anion transporters, and green tea intake was found to increase systemic levels of endogenous indoxyl sulfate and *p*-cresyl sulfate and levels of serum creatinine and blood urea nitrogen in rats with adenine-induced chronic renal failure (Peng et al., 2015). The authors concluded that green tea metabolites impair renal function by inhibiting the uptake transporting functions of OAT1 and OAT3, thereby reducing the elimination of nephro-cardiovascular toxins.

## Conclusions

**Figure 5:**
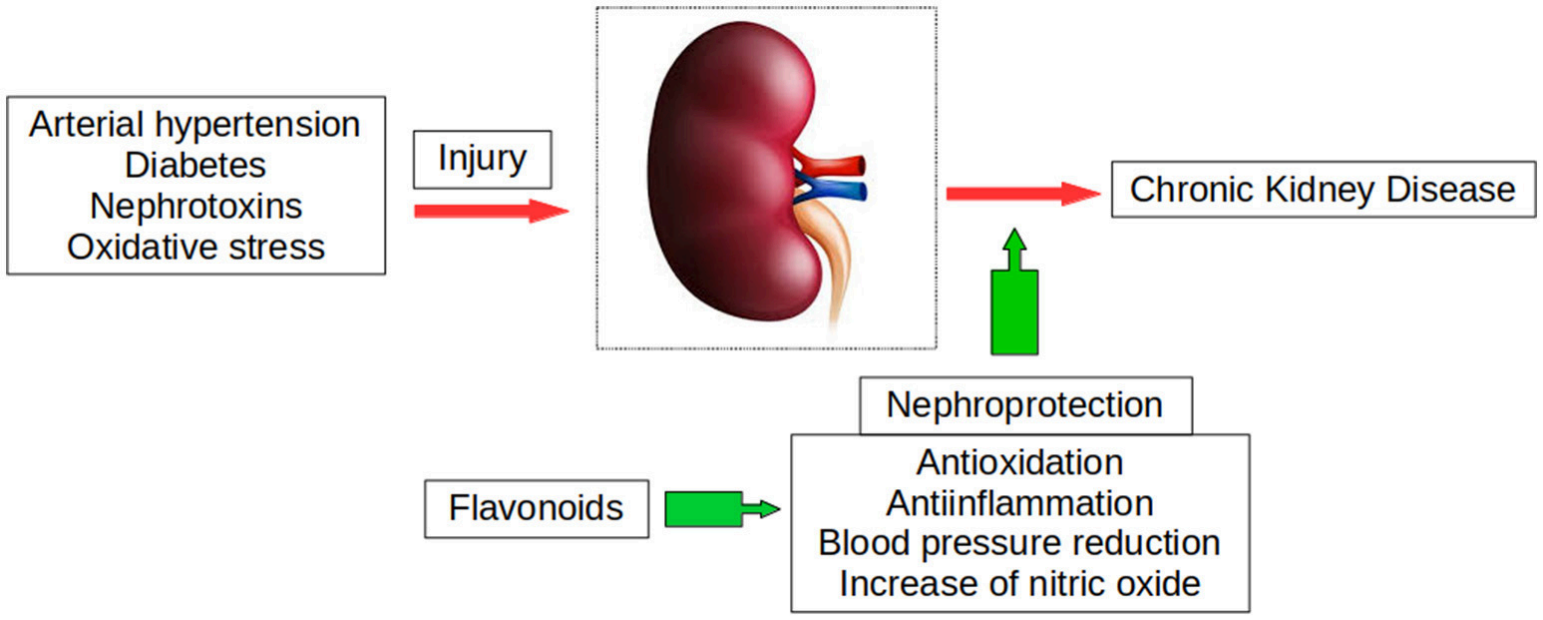
Flavonoids exert renoprotective actions due to their multiple beneficial properties, counteracting the deleterious effects of kidney injuries that can lead to chronic kidney disease.

The studies in this review demonstrate that flavonoids have important effects on renal physiology and possess diuretic and natriuretic properties, as well as exerting renoprotective effects in AKI and CKD of different etiologies (Figure 5). Their protective effects against the different nephropathies reviewed indicate that their main mechanism of action is their impact on the inflammatory cascade and oxidative perturbations, affecting several pathways. Flavonoids also possess major antiapoptotic and antifibrotic properties, inhibiting EMT and interfering with TGF-β1/Smad signaling, which may play an essential role in their renoprotective action in CKD. Various flavonoids also develop antitumor activity in renal carcinoma cells, inducing cell cycle arrest and apoptotic cell death *via* several pathways and suppressing renal carcinoma cell proliferation *in vitro* and *in vivo*. These antitumor effects are associated with scant toxicity in normal cells.

## Perspectives

CKD is a public health epidemic associated with an increased risk of death and CVD, including uremic cardiomyopathy and vascular calcification. This review of recent advances in our understanding of the effects and action mechanisms of flavonoids show that they can attenuate the AKI induced by different nephrotoxic agents and reduce renal fibrosis. These data support the possible usefulness of these compounds as novel therapeutic agents against chronic complications in patients, including CVD in CKD. However, numerous methodological problems prevent the drawing of exhaustive and clear conclusions on this issue, which warrants considerable further research. The development of new therapeutic strategies requires a greater understanding of the cellular and molecular mechanisms underlying the effect of flavonoids on CKD progression and CVD development. There is a need to clearly establish and standardize the composition of compounds in polyphenols in order to elucidate and reproduce their therapeutic mechanisms of action. In addition, it is important to identify the metabolites resulting from their administration, which may also be responsible for their effects. Translational investigations are also required to enhance bench-to-bedside transition, exploring whether polyphenols and flavonoids can become pharmacological agents of natural origin to improve the treatment of cardiovascular and renal diseases.

## Author contributions

RW and PV-T searched and contributed the first draft of Sections Nephrotoxic Agents, Ischemia-Reperfusion Injury and Other Surgical Procedures, Chronic Kidney Disease, Renal Apoptosis and Fibrosis, and Renal Carcinoma. They also made Figures 4 and 5 and the corresponding list of references. PR and AG-G made a similar job with Sections Renal Physiology, Hypertensive Nephropathy, and Obesity and Diabetes, Figures 1–3 and references. NA and JG-E worked with this part of the review. PV-T prepared the final draft combining both parts. MP revised english grammar and typographical errors. FV and JG-E approved the final version, obtained feedback and consent from every single author.

### Conflict of interest statement

The authors declare that the research was conducted in the absence of any commercial or financial relationships that could be construed as a potential conflict of interest.

## Abbreviations

AKI: acute kidney injury
CKD: chronic kidney disease
CVD: cardiovascular disease
CRP: C-reactive protein
DOCA-salt: deoxicorticosterone acetate- salt
ENaC: epithelial Na^+^ channel
EMT: epithelial to mesenchymal transition
GSH: glutathione
IL: interleukin
I/R: ischemia/reperfusion
JNK: janus kinase
L-NAME: Nω- nitro-L-arginine methyl ester
LPS: lipopolysaccharide
MAPK: mitogen-activated protein kinase
NKCC1: Na^+^-K^+^-2Cl^−^ cotransporter 1
NO: nitric oxide
NOS: nitric oxide synthase
ROS: reactive oxygen species
SHR: spontaneously hypertensive rats
TBARS: thiobarbituric acid reactive substances
STZ: streptozotocin
TLR-4: Toll-like receptor 4
TNF-α: tumor necrosis factor-α.

## References

[B1] Ali B. H. Al Za'abi M. Adham S. A. Yasin J. Nemmar A. Schupp N. (2016). Therapeutic effect of chrysin on adenine-induced chronic kidney disease in rats. Cell. Physiol. Biochem. 38, 248–257. 10.1159/000438626 26784294

[B2] Aoi W. Niisato N. Miyazaki H. Marunaka Y. (2004). Flavonoid-induced reduction of ENaC expression in the kidney of Dahl salt-sensitive hypertensive rat. Biochem. Biophys. Res. Commun. 315, 892–896. 10.1016/j.bbrc.2004.01.150 14985096

[B3] Arab H. H. Mohamed W. R. Barakat B. M. Arafa-el S. A. (2016). Tangeretin attenuates cisplatin-induced renal injury in rats: impact on the inflammatory cascade and oxidative perturbations. Chem. Biol. Interact. 258, 205–213. 10.1016/j.cbi.2016.09.008 27616468

[B4] Bagheri F. Gol A. Dabiri S. Javadi A. (2011). Preventive effect of garlic juice on renal reperfusion injury. Iran J. Kidney Dis. 5, 194–200. 21525580

[B5] Bai S. Huang Z. G. Chen L. Wang J. T. Ding B. P. (2013). Effects of felodipine combined with puerarin on ACE2-Ang (1-7)-Mas axis in renovascular hypertensive rat. Regul. Pept. 184, 54–61. 10.1016/j.regpep.2013.03.005 23523569

[B6] Bhalodia Y. Kanzariya N. Patel R. Patel N. Vaghasiya J. Jivani R. H. (2009). Renoprotective activity of *Benincasa Cerifera* fruit extracton ischemia/reperfusion-induced renal damage in rat. Iran J. Kidney Dis. 3, 80–85. 19395782

[B7] Cao J. Wang H. Chen F. Fang J. Xu A. Xi W.. (2016). Galangin inhibits cell invasion by suppressing the epithelial-mesenchymal transition and inducing apoptosis in renal cell carcinoma. Mol. Med. Rep. 13, 4238–4244. 10.3892/mmr.2016.5042 27035542 PMC4838127

[B8] Cesarone M. R. Belcaro G. Stuard S. Schönlau F. Di Renzo A. Grossi M. G.. (2010). Kidney flow and function in hypertension: protective effects of pycnogenol in hypertensive participants-a controlled study. J. Cardiovasc. Pharmacol. Ther. 15, 41–46. 10.1177/1074248409356063 20097689

[B9] Chao C. S. Tsai C. S. Chang Y. P. Chen J. M. Chin H. K. Yang S. C. (2016). Hyperin inhibits nuclear factor kappa B and activates nuclear factor E2-related factor-2 signaling pathways in cisplatin-induced acute kidney injury in mice. Int. Immunopharmacol. 40, 517–523. 10.1016/j.intimp.2016.09.020 27764742

[B10] Chen G. Tan M. L. Li K. K. Leung P. C. Ko C. H. (2015). Green tea polyphenols decreases uric acid level through xanthine oxidase and renal urate transporters in hyperuricemic mice. J. Ethnopharmacol. 175, 14–20 10.1016/j.jep.2015.08.043 26344851

[B11] Chen J. Du L. Li J. Song H. (2016). Epigallocatechin-3-gallate attenuates cadmium-induced chronic renal injury and fibrosis. Food Chem. Toxicol. 96, 70–78. 10.1016/j.fct.2016.07.030 27474435

[B12] Coresh J. Selvin E. Stevens L. A. Manzi J. Kusek J. W. Eggers P.. (2007). Prevalence of chronic kidney disease in the United States. JAMA. 298, 2038–2047. 10.1001/jama.298.17.2038 17986697

[B13] de Almeida C. L. Boeing T. Somensi L. B. Steimbach V. M. da Silva L. M. Andrade S. F.. (2017). Diuretic, natriuretic and potassium-sparing effect of nothofagin isolated from Leandra dasytricha (A. Gray) Cogn. leaves in normotensive and hypertensive rats. Chem. Biol. Interact. 268, 103–110. 10.1016/j.cbi.2017.03.004 28284659

[B14] de Souza P. Boeing T. Somensi L. B. Cechinel-Zanchett C. C. Bastos J. K. Petreanu M. (2017). Diuretic effect of extracts, fractions and two compounds 2α,3β,19α-trihydroxy-urs-12-en-28-oic acid and 5-hydroxy-3,6,7,8,4'-pentamethoxyflavone from *Rubus rosaefolius* Sm. (Rosaceae) leaves in rats. Naunyn Schmiedebergs. Arch. Pharmacol. 390, 351–360. 10.1007/s00210-016-1333-4 28013356

[B15] Duarte J. Jiménez R. O'Valle F. Galisteo M. Perez-Palencia R. Vargas F.. (2002). Protective effects of the flavonoid quercetin in chronic nitric oxide deficient rats. J. Hypertens. 20, 1843–1854. 10.1097/00004872-200209000-00031 12195128

[B16] Duarte J. Pérez-Palencia R. Vargas F. Ocete M. A. Pérez-Vizcaino F. Zarzuelo A.. (2001). Antihypertensive effects of the flavonid quercetin in spontaneously hypertensive rats. Br. J. Pharmacol. 133, 117–124. 10.1038/sj.bjp.0704064 11325801 PMC1572775

[B17] El-Kashef D. H. El-Kenawi A. E. Suddek G. M. Salem H. A. (2015), Flavocoxid attenuates gentamicin-induced nephrotoxicity in rats. Naunyn Schmiedebergs Arch. Pharmacol. 388, 1305–1315. 10.1007/s00210-015-1164-8 26272642

[B18] Eo H. Park J. E. Jeon Y. J. Lim Y. (2017). Ameliorative effect of ecklonia cava polyphenol extract on renal inflammation associated with aberrant energy metabolism and oxidative stress in high fat diet-induced obese mice. J. Agric. Food Chem. 65, 3811–3818. 10.1021/acs.jafc.7b00357 28459555

[B19] Erboga M. Aktas C. Erboga Z. F. Donmez Y. B. Gurel A. (2015). Quercetin ameliorates methotrexate-induced renal damage, apoptosis and oxidative stress in rats. Ren. Fail. 37, 1492–1497. 10.3109/0886022X.2015.1074521 26338102

[B20] Funamoto M. Masumoto H. Takaori K. Taki T. Setozaki S. Yamazaki K.. (2016). Green tea polyphenol prevents diabetic rats from acute fidney injury after cardiopulmonary bypass. Ann. Thorac. Surg. 101, 1507–1513. 10.1016/j.athoracsur.2015.09.080 26675556

[B21] Galisteo M. García-Saura M. F. Jiménez R. Villar I. C. Wangensteen R. Zarzuelo A.. (2004b). Effects of quercetin treatment on vascular function in deoxycorticosterone acetate-salt hypertensive rats. Comparative study with verapamil. Planta Med. 70, 334–341. 10.1055/s-2004-818945 15095149

[B22] Galisteo M. García-Saura M. F. Jiménez R. Villar I. C. Zarzuelo A. Vargas F.. (2004a). Effects of chronic quercetin treatment on antioxidant defence system and oxidative status of deoxycorticosterone acetate-salt-hypertensive rats. Mol. Cell. Biochem. 259, 91–99. 10.1023/B:MCBI.0000021360.89867.64 15124912

[B23] Gao Z. Han Y. Hu Y. Wu X. Wang Y. Zhang X.. (2016). Targeting HO-1 by epigallocatechin-3-gallate reduces contrast-induced renal injury via anti-oxidative stress and anti-inflammation pathways. PLoS ONE 11:e0149032. 10.1371/journal.pone.0149032 26866373 PMC4750900

[B24] García-Saura M. F. Galisteo M. Villar I. C. Bermejo A. Zarzuelo A. Vargas F.. (2005). Effects of chronic quercetin treatment in experimental renovascular hypertension. Mol. Cell. Biochem. 270, 147–155. 10.1007/s11010-005-4503-0 15792364

[B25] Gómez-Guzmán M. Jiménez R. Sánchez M. Zarzuelo M. J. Galindo P. Quintela A. M.. (2012). Epicatechin lowers blood pressure, restores endothelial function and decreases oxidative stress, endothelin-1 and NADPH oxidase activity in DOCA-salt hypertension. Free Radic. Biol. Med. 52, 70–79. 10.1016/j.freeradbiomed.2011.09.015 22001745

[B26] Han M. A. Lee D. H. Woo S. M. Seo B. R. Min K. J. Kim S.. (2016). Galangin sensitizes TRAIL-induced apoptosis through down-regulation of anti-apoptotic proteins in renal carcinoma Caki cells. Sci. Rep. 6:18642. 10.1038/srep18642 26725939 PMC4698673

[B27] Han Y. Lu J. S. Xu Y. Zhang L. Hong B. F. (2015). Rutin ameliorates renal fibrosis and proteinuria in 5/6-nephrectomized rats by anti-oxidation and inhibiting activation of TGFβ1-smad signaling. Int. J. Clin. Exp. Pathol. 8, 4725–4734. 26191162 PMC4503034

[B28] Hassan S. M. Khalaf M. M. Sadek S. A. Abo-Youssef A. M. (2017). Protective effects of apigenin and myricetin against cisplatin-induced nephrotoxicity in mice. Pharm. Biol. 55, 766–774. 10.1080/13880209.2016.1275704 28064632 PMC6130592

[B29] He X. Li C. Wei Z. Wang J. Kou J. Liu W.. (2016). Protective role of apigenin in cisplatin-induced renal injury. Eur. J. Pharmacol. 789, 215–221. 10.1016/j.ejphar.2016.07.003 27388142

[B30] Huang D. Wang C. Duan Y. Meng Q. Liu Z. Huo X.. (2017). Targeting Oct2 and P53: formononetin prevents cisplatin-induced acute kidney injury. Toxicol. Appl. Pharmacol. 326, 15–24. 10.1016/j.taap.2017.04.013 28414026

[B31] Huang Y. C. Tsai M. S. Hsieh P. C. Shih J. H. Wang T. S. Wang Y. C.. (2017). Galangin ameliorates cisplatin-induced nephrotoxicity by attenuating oxidative stress, inflammation and cell death in mice through inhibition of ERK and NF-kappaB signaling. Toxicol. Appl. Pharmacol. 329, 128–139. 10.1016/j.taap.2017.05.034 28558962

[B32] Huang Z. He L. Huang D. Lei S. Gao J. (2015). Icariin protects rats against 5/6 nephrectomy-induced chronic kidney failure by increasing the number of renal stem cells. BMC Complement. Altern. Med. 15:378. 10.1186/s12906-015-0909-8 26490949 PMC4617909

[B33] Jiménez R. López-Sepúlveda R. Kadmiri M. Romero M. Vera R. Sánchez M.. (2007). Polyphenols restore endothelial function in DOCA-salt hypertension: role of endothelin-1 and NADPH oxidase. Free Radic. Biol. Med. 43, 462–473. 10.1016/j.freeradbiomed.2007.05.007 17602962

[B34] Kabasakal L. Sehirli O. Cetinel S. Cikler E. Gedik N. Sener G. (2005). Protective effect of aqueous garlic extract against renal ischemia/reperfusion injury in rats. J. Med. Food 8, 319–326. 10.1089/jmf.2005.8.319 16176141

[B35] Kang M. K. Park S. H. Choi Y. J. Shin D. Kang Y. H. (2015). Chrysin inhibits diabetic renal tubulointerstitial fibrosis through blocking epithelial to mesenchymal transition. J. Mol. Med. 93, 759–772. 10.1007/s00109-015-1301-3 26062793

[B36] Katary M. Salahuddin A. (2017). Ameliorative effect of gossypin against gentamicin-induced nephrotoxicity in rats. Life Sci. 176, 75–81. 10.1016/j.lfs.2017.03.009 28302561

[B37] Kaur S. Muthuraman A. (2016). Therapeutic evaluation of rutin in two-kidney one-clip model of renovascular hypertension in rat. Life Sci. 150, 89–94. 10.1016/j.lfs.2016.02.080 26920631

[B38] Lan C. Z. Ding L. Su Y. L. Guo K. Wang L. Kan H. W.. (2015). Grape seed proanthocyanidins prevent DOCA-salt hypertension-induced renal injury and its mechanisms in rats. Food Funct. 6, 2179–2186. 10.1039/C5FO00253B 26011796

[B39] Lee I. C. Ko J. W. Park S. H. Shin N. R. Shin I. S. Kim Y. B.. (2017). Ameliorative effects of pine bark extract on cisplatin-induced acute kidney injury in rats. Ren. Fail. 39, 363–371. 10.1080/0886022X.2017.1282871 28178874 PMC6014499

[B40] Li W. Li H. Zhang M. Wang M. Zhong Y. Wu H.. (2016). Quercitrin ameliorates the development of systemic lupus erythematosus-like disease in a chronic graft-versus-host murine model. Am. J. Physiol. Renal Physiol. 311, F217–F226. 10.1152/ajprenal.00249.2015 26911849

[B41] Li W. Wang L. Chu X. Cui H. Bian Y. (2017). Icariin combined with human umbilical cord mesenchymal stem cells significantly improve the impaired kidney function in chronic renal failure. Mol. Cell Biochem. 428, 203–212. 10.1007/s11010-016-2930-8 28116543

[B42] Liao J. Liu Y. Wu H. Zhao M. Tan Y. Li D.. (2016). The role of icaritin in regulating Foxp3/IL17a balance in systemic lupus erythematosus and its effects on the treatment of MRL/lpr mice. Clin. Immunol. 162, 74–83. 10.1016/j.clim.2015.11.006 26604013

[B43] Lin M. Li L. Li L. Pokhrel G. Qi G. Rong R.. (2014). The protective effect of baicalin against renal ischemia-reperfusion injury through inhibition of inflammation and apoptosis. BMC Complement. Altern. Med. 14:19. 10.1186/1472-6882-14-19 24417870 PMC3893527

[B44] Liu C. Kang Y. Zhou X. Yang Z. Gu J. Han C. (2017). Rhizoma smilacis glabrae protects rats with gentamicin-induced kidney injury from oxidative stress-induced apoptosis by inhibiting caspase-3 activation. J. Ethnopharmacol. 198, 122–130. 10.1016/j.jep.2016.12.034 28034658

[B45] Liu Y. An W. Gao A. (2016). Protective effects of naringenin in cardiorenal syndrome. J. Surg. Res. 203, 416–423. 10.1016/j.jss.2016.03.003 27363651

[B46] Lv J. Feng M. Zhang L. Wan X. Zeng Y. C. Liang P. F.. (2015). Protective effect of epigallocatechin gallate, a major constituent of green tea, against renal ischemia-reperfusion injury in rats. Int. Urol. Nephrol. 47, 1429–1435. 10.1007/s11255-015-1030-0 26122117 PMC4518080

[B47] Ma X. Yan L. Zhu Q. Shao F. (2017). Puerarin attenuates cisplatin-induced rat nephrotoxicity: the involvement of TLR4/NF-κB signaling pathway. PLoS ONE 12:e0171612. 10.1371/journal.pone.0171612 28182789 PMC5300759

[B48] Ma Z. Liu W. Zeng J. Zhou J. Guo P. Xie H.. (2015). Silibinin induces apoptosis through inhibition of the mTOR-GLI1-BCL2 pathway in renal cell carcinoma. Oncol. Rep. 34, 2461–2468. 10.3892/or.2015.4224 26323996

[B49] Malik S. Bhatia J. Suchal K. Gamad N. Dinda A. K. Gupta Y. K.. (2015). Nobiletin ameliorates cisplatin-induced acute kidney injury due to its anti-oxidant, anti-inflammatory and anti-apoptotic effects. Exp. Toxicol. Pathol. 67, 427–433. 10.1016/j.etp.2015.04.008 26002685

[B50] Malik S. Suchal K. Bhatia J. Gamad N. Dinda A. K. Gupta Y. K.. (2016). Molecular mechanisms underlying attenuation of cisplatin-induced acute kidney injury by epicatechin gallate. Lab. Invest. 96, 853–861. 10.1038/labinvest.2016.60 27239733

[B51] Malik S. Suchal K. Khan S. I. Bhatia J. Kishore K. Dinda A. K.. (2017). Apigenin ameliorates streptozotocin-induced diabetic nephropathy in rats via MAPK-NF-κB-TNF-α and TGF-β1-MAPK-fibronectin pathways. Am. J. Physiol. Renal Physiol. 313, F414–F422. 10.1152/ajprenal.00393.2016 28566504

[B52] Manach C. Scalbert A. Morand C. Rémésy C. Jiménez L. (2004). Polyphenols: food sources and bioavailability. Am. J. Clin. Nutr. 79, 727–747. 10.1093/ajcn/79.5.727 15113710

[B53] Marunaka Y. (2017). Actions of quercetin, a flavonoid, on ion transporters: its physiological roles. Ann. N.Y. Acad. Sci. 1398, 142–151. 10.1111/nyas.13361 28574574

[B54] Meng F. D. Li Y. Tian X. Ma P. Sui C. G. Fu L. Y.. (2015). Synergistic effects of snail and quercetin on renal cell carcinoma Caki-2 by altering AKT/mTOR/ERK1/2 signaling pathways. Int. J. Clin. Exp. Pathol. 8, 6157–6168. 26261493 PMC4525827

[B55] Meng S. Zhu Y. Li J. F. Wang X. Liang Z. Li S. Q.. (2017). Apigenin inhibits renal cell carcinoma cell proliferation. Oncotarget 8, 19834–19842. 10.18632/oncotarget.15771 28423637 PMC5386726

[B56] Mezesova L. Bartekova M. Javorkova V. Vlkovicova J. Breier A. Vrbjar N. (2010). Effect of quercetin on kinetic properties of renal Na,K-ATPase in normotensive and hypertensive rats. J. Physiol. Pharmacol. 61, 593–598. 21081803

[B57] Middleton E. Jr. Kandaswami C. Theoharides T. C. (2000). The effects of plant flavonoids on mammalian cells: implications for inflammation, heart disease, and cancer. Pharmacol. Rev. 52, 673–751. 11121513

[B58] Mohan T. Velusamy P. Chakrapani L. N. Srinivasan A. K. Singh A. Johnson T.. (2017). Impact of EGCG supplementation on the progression of diabetic nephropathy in rats: an insight into fibrosis and apoptosis. J. Agric. Food Chem. 65, 8028–8036. 10.1021/acs.jafc.7b03301 28823168

[B59] Palabiyik S. S. Dincer B. Cadirci E. Cinar I. Gundogdu C. Polat B.. (2017). A new update for radiocontrast-induced nephropathy aggravated with glycerol in rats: the protective potential of epigallocatechin-3-gallate. Ren. Fail. 39, 314–322. 10.1080/0886022X.2016.1277245 28100100 PMC6014352

[B60] Palanisamy N. Venkataraman A. C. (2013). Beneficial effect of genistein on lowering blood pressure and kidney toxicity in fructose-fed hypertensive rats. Br. J. Nutr. 109, 1806–1812. 10.1017/S0007114512003819 23116847

[B61] Peng Y. H. Sweet D. H. Lin S. P. Yu C. P. Lee Chao P. D. Hou Y. C. (2015). Green tea inhibited the elimination of nephro-cardiovascular toxins and deteriorated the renal function in rats with renal failure. Sci. Rep. 5:16226. 10.1038/srep16226 26552961 PMC4639770

[B62] Prahan P. Kumar S. Raja B. (2012). Effect of morin, a flavonoid against DOCA-salt hypertensive rats: a dose dependent study. Asian Pac. J. Trop. Biomed. 2, 443–448. 10.1016/S2221-1691(12)60073-2 23569947 PMC3609328

[B63] Pribac G. C. Sferdian M. F. Neamtu C. Crăciun C. Roşioru C. L. Ardelean A.. (2015). Fenugreek powder exerts protective effects on alcoholised rats' kidney, highlighted using ultrastructural studies. Rom. J. Morphol. Embryol. 56, 445–451. 26193212

[B64] Prince P. D. Fischerman L. Toblli J. E. Fraga C. G. Galleano M. (2017). LPS-induced renal inflammation is prevented by (-)-epicatechin in rats. Redox. Biol. 11, 342–349. 10.1016/j.redox.2016.12.023 28039839 PMC5200882

[B65] Prince P. D. Lanzi C. R. Toblli J. E. Elesgaray R. Oteiza P. I. Fraga C. G.. (2016). Dietary (-)-epicatechin mitigates oxidative stress, NO metabolism alterations, and inflammation in renal cortex from fructose-fed rats. Free Radic. Biol. Med. 90, 35–46. 10.1016/j.freeradbiomed.2015.11.009 26569027

[B66] Promsan S. Jaikumkao K. Pongchaidecha A. Chattipakorn N. Chatsudthipong V. Arjinajarn P.. (2016). Pinocembrin attenuates gentamicin-induced nephrotoxicity in rats. Can. J. Physiol. Pharmacol. 94, 808–818. 10.1139/cjpp-2015-0468 27245556

[B67] Rassaf T. Rammos C. Hendgen-Cotta U. B. Heiss C. Kleophas W. Dellanna F.. (2016). Vasculoprotective effects of dietary cocoa flavanols in patients on hemodialysis: a double-blind, randomized, placebo-controlled trial. Clin. J. Am. Soc. Nephrol. 11, 108–118 10.2215/CJN.05560515 26681132 PMC4702234

[B68] Romero M. Jiménez R. Hurtado B. Moreno J. M. Rodríguez-Gómez I. López-Sepúlveda R.. (2010). Lack of beneficial metabolic effects of quercetin in adult spontaneously hypertensive rats. Eur. J. Pharmacol. 627, 242–250. 10.1016/j.ejphar.2009.11.006 19903466

[B69] Roy S. Ahmed F. Banerjee S. Saha U. (2016). Naringenin ameliorates streptozotocin-induced diabetic rat renal impairment by downregulation of TGF-β1 and IL-1 via modulation of oxidative stress correlates with decreased apoptotic events. Pharm. Biol. 54, 1616–1627. 10.3109/13880209.2015.1110599 26928632

[B70] Sengupta B. Sahihi M. Dehkhodaei M. Kelly D. Arany I. (2017). Differential roles of 3-Hydroxyflavone and 7-Hydroxyflavone against nicotine-induced oxidative stress in rat renal proximal tubule cells. PLoS ONE 12:e0179777. 10.1371/journal.pone.0179777 28640852 PMC5480997

[B71] Tanabe K. Tamura Y. Lanaspa M. A. Miyazaki M. Suzuki N. Sato W.. (2012). Epicatechin limits renal injury by mitochondrial protection in cisplatin nephropathy. Am. J. Physiol. Renal Physiol. 303, F1264–F1274. 10.1152/ajprenal.00227.2012 22933302 PMC5243204

[B72] Tang Y. Choi E. J. Han W. C. Oh M. Kim J. Hwang J. Y.. (2017). Moringa oleifera from cambodia ameliorates oxidative stress, hyperglycemia, and kidney dysfunction in type 2 diabetic mice. J. Med. Food. 20, 502–510. 10.1089/jmf.2016.3792 28467233

[B73] Tian W. Lei H. Guan R. Xu Y. Li H. Wang L.. (2015). Icariside II ameliorates diabetic nephropathy in streptozotocin-induced diabetic rats. Drug Des. Devel. Ther. 9, 5147–5157. 10.2147/DDDT.S90060 26379427 PMC4567177

[B74] Van Berendoncks A. M. Elseviers M. M. Lins R. L. (2010). Outcome of acute kidney injury with different treatment options: long-term follow-up. Clin. J. Am. Soc. Nephrol. 5, 1755–1762. 10.2215/CJN.00770110 20634328 PMC2974373

[B75] Wang B. Liu D. Zhu Q. H. Li M. Chen H. Guo Y.. (2016). Rutin ameliorates kidney interstitial fibrosis in rats with obstructive nephropathy. Int. Immunopharmacol. 35, 77–84. 10.1016/j.intimp.2016.03.029 27035719

[B76] Wang H. Li D. Hu Z. Zhao S. Zheng Z. Li W. (2016). Protective effects of green tea polyphenol against renal injury through ROS-mediated JNK-MAPK pathway in lead exposed rats. Mol. Cells. 39, 508–513. 10.14348/molcells.2016.2170 27239812 PMC4916403

[B77] Wang Q. Z. Gao H. Q. Liang Y. Zhang J. Wang J. Qiu J. (2015). Cofilin1 is involved in hypertension-induced renal damage via the regulation of NF-κB in renal tubular epithelial cells. J. Transl. Med. 13:323. 10.1186/s12967-015-0685-8 26450610 PMC4599745

[B78] Wang X. Zhao X. Feng T. Jin G. Li Z. (2016). Rutin prevents high glucose-induced renal glomerular endothelial hyperpermeability by inhibiting the ROS/Rhoa/ROCK signaling pathway. Planta Med. 82, 1252–1257. 10.1055/s-0042-110859 27552253

[B79] Wei X. Gao P. Pu Y. Li Q. Yang T. Zhang H.. (2017). Activation of TRPV4 by dietary apigenin antagonizes renal fibrosis in deoxycorticosterone acetate (DOCA)-salt-induced hypertension. Clin. Sci. 131, 567–581. 10.1042/CS20160780 28143892

[B80] Williams R. J. Spencer J. P. Rice-Evans C. (2004). Flavonoids: antioxidants or signalling molecules? Free Radic. Biol. Med. 36, 838–849. 10.1016/j.freeradbiomed.2004.01.001 15019969

[B81] Woo S. M. Kwon T. K. (2016). Jaceosidin induces apoptosis through Bax activation and down-regulation of Mcl-1 and c-FLIP expression in human renal carcinoma Caki cells. Chem. Biol. Interact. 260, 168–175. 10.1016/j.cbi.2016.10.011 27729209

[B82] Xin S. B. Yan H. Ma J. Sun Q. Shen L. (2016). Protective effects of luteolin on lipopolysaccharide-induced acute renal injury in mice. Med. Sci. Monit. 22, 5173–5180. 10.12659/MSM.898177 28029146 PMC5215379

[B83] Xu X. Zheng N. Chen Z. Huang W. Liang T. Kuang H. (2016). Puerarin, isolated from Pueraria lobata (Willd.), protects against diabetic nephropathy by attenuating oxidative stress. Gene. 591, 411–416. 10.1016/j.gene.2016.06.032 27317894

[B84] Yang H. Zuo X. Z. Tian C. He D. L. Yi W. J. Chen Z.. (2015). Green tea polyphenols attenuate high-fat diet-induced renal oxidative stress through SIRT3-dependent deacetylation. Biomed. Environ. Sci. 28, 455–459. 10.3967/bes2015.064 26177907

[B85] Yang K. Li W. F. Yu J. F. Yi C. Huang W. F. (2017). Diosmetin protects against ischemia/reperfusion-induced acute kidney injury in mice. J. Surg. Res. 214, 69–78. 10.1016/j.jss.2017.02.067 28624062

[B86] Yang X. H. Pan Y. Zhan X. L. Zhang B. L. Guo L. L. Jin H. M. (2016). Epigallocatechin-3-gallate attenuates renal damage by suppressing oxidative stress in diabetic db/db mice. Oxid. Med. Cell Longev. 2016:2968462. 10.1155/2016/2968462 27698952 PMC5028863

[B87] Ye J. H. Liu M. H. Zhang X. L. He J. Y. (2015). Chemical profiles and protective effect of hedyotis diffusa willd in lipopolysaccharide-induced renal inflammation mice. Int. J. Mol. Sci. 16, 27252–27269. 10.3390/ijms161126021 26580602 PMC4661879

[B88] Zhang J. Fu H. Xu Y. Niu Y. An X. (2016). Hyperoside reduces albuminuria in diabetic nephropathy at the early stage through ameliorating renal damage and podocyte injury. J. Nat. Med. 70, 740–748. 10.1007/s11418-016-1007-z 27255369

[B89] Zhang Z. Gao X. Guo M. Jiang H. Cao Y. Zhang N. (2017). The protective effect of baicalin against lead-induced renal oxidative damage in mice. Biol. Trace Elem. Res. 175, 129–135. 10.1007/s12011-016-0731-2 27209023

[B90] Zhao L. Xu L. Tao X. Han X. Yin L. Qi Y.. (2016). Protective effect of the total flavonoids from *Rosa laevigata* michx fruit on renal ischemia-reperfusion injury through suppression of oxidative stress and inflammation. Molecules. 21:E952. 10.3390/molecules21070952 27455216 PMC6272996

[B91] Zheng L. Zhang C. Li L. Hu C. Hu M. Sidikejiang N.. (2017). Baicalin ameliorates renal fibrosis via inhibition of transforming growth factor β1 production and downstream signal transduction. Mol. Med. Rep. 15, 1702–1712. 10.3892/mmr.2017.6208 28260014 PMC5364985

[B92] Zhou X. Bai C. Sun X. Gong X. Yang Y. Chen C.. (2017). Puerarin attenuates renal fibrosis by reducing oxidative stress induced-epithelial cell apoptosis via MAPK signal pathways *in vivo* and *in vitro*. Ren. Fail. 39, 423–431. 10.1080/0886022X.2017.1305409 28335679 PMC6014507

[B93] Zhu Y. Fu Y. Lin H. (2016). Baicalin inhibits renal cell apoptosis and protects against acute kidney injury in pediatric sepsis. Med. Sci. Monit. 22, 5109–5115. 10.12659/MSM.899061 28013315 PMC5207012

